# Morphine Promotes Tumor Angiogenesis and Increases Breast Cancer Progression

**DOI:** 10.1155/2015/161508

**Published:** 2015-05-03

**Authors:** Sabrina Bimonte, Antonio Barbieri, Domenica Rea, Giuseppe Palma, Antonio Luciano, Arturo Cuomo, Claudio Arra, Francesco Izzo

**Affiliations:** Istituto Nazionale per lo Studio e la Cura dei Tumori “Fondazione G. Pascale”, IRCCS, Via Mariano Semmola, 80131 Naples, Italy

## Abstract

Morphine is considered a highly potent analgesic agent used to relieve suffering of patients with cancer. Several *in vitro* and *in vivo* studies showed that morphine also modulates angiogenesis and regulates tumour cell growth. Unfortunately, the results obtained by these studies are still contradictory. In order to better dissect the role of morphine in cancer cell growth and angiogenesis we performed *in vitro* studies on ER-negative human breast carcinoma cells, MDA.MB231 and *in vivo* studies on heterotopic mouse model of human triple negative breast cancer, TNBC. We demonstrated that morphine *in vitro* enhanced the proliferation and inhibited the apoptosis of MDA.MB231 cells. *In vivo* studies performed on xenograft mouse model of TNBC revealed that tumours of mice treated with morphine were larger than those observed in other groups. Moreover, morphine was able to enhance the neoangiogenesis. Our data showed that morphine at clinical relevant doses promotes angiogenesis and increases breast cancer progression.

## 1. Introduction

Morphine is an opiate-based drug largely used to relieve pains of patients with cancer in terminal phases, in order to improve their quality of life [1]. It was isolated for the first time in 1803 by Friedrich W. Sertürner [2]. It is noted that morphine explains its function by acting through opioid receptors localized in the brain named *μ*, *δ*, and *κ* [3, 4]. Morphine relieves pain by acting directly on central nervous system (CNS), although its activity on peripheral tissue leads to many secondary complications, including immunocostipation, respiratory depression, addiction, and tolerance. Morphine is still considered the most effective analgesic clinically available used to relieve suffering of patients with cancer [5]. Several experimental studies performed on cancer cell lines and mouse models showed that morphine can also play a role in regulation of cancer cell growth. Unfortunately, at present the role of morphine in the regulation of tumor cell growth is not yet correctly established. The results obtained by these studies are still contradictory. Many reports showed that morphine was able to inhibit the growth of various human cancer cell lines [6–12] or animal models [13–16]. On the contrary, other studies proved that morphine increased tumor cell growth in* in vivo* [17, 18] or* in vitro* [19] models. It has been demonstrated that morphine at clinically relevant doses stimulates angiogenesis* in vitro* [20], promotes tumour growth in breast cancer mouse model, and increases vascular permeability [21]. One explanation for these different results could be due to different concentration and/or time of administration of morphine applied. In fact,* in vitro* and* in vivo* studies showed that tumor suppression occurs after chronic high doses of morphine [11, 15, 16], while tumor-enhancing effects with morphine occur after administration of low daily doses or single dose of morphine [22]. Thus, there is a dilemma about the effects of morphine on cancer cell growth and angiogenesis [23]. Recently, it has been demonstrated that morphine stimulates cancer progression and mast cell activation and impairs survival in transgenic mice with breast cancer [24].

For these reasons, in order to elucidate the role of morphine in regulation of tumor growth and angiogenesis in triple negative breast cancer (TNBC), we performed* in vitro* and* in vivo *studies on the ER-negativehuman breast carcinoma cells MDA.MB231. Our data showed that morphine at clinical relevant doses promotes tumor angiogenesis and increases breast cancer proliferation and migration.

## 2. Materials and Methods

### 2.1. Materials

Morphine sulphate used for* in vitro *and* in vivo *experiments was kindly gifted by Dr. Arturo Cuomo (IRCCS Fondazione Pascale) and was dissolved in distilled water to a concentration of 100 mM as a stock solution. Then the drug was added to MDA.MB231 cells in three different doses (1, 10, and 100 *μ*M). The antibody against PECAM-1 was obtained from Santa Cruz Biotechnology (Santa Cruz, CA). Anti-p53 antibody was kindly provided by Imgenex (San Diego, CA). The liquid DAB+ Substrate Chromogen System-HRP used for immunocytochemistry was obtained from DakoCytomation (Carpinteria, CA). Penicillin, streptomycin, Dulbecco's modified Eagle medium (DMEM), and fetal bovine serum (FBS) were obtained from Invitrogen (Grand Island, NY). Tris, glycine, NaCl, SDS, and bovine serum albumin (BSA) were obtained from Sigma Chemical (St. Louis, MO).

### 2.2. Cell Lines

ER-negativebreast cancer cell line MDA.MB231 was obtained from American Type Culture Collection (Manassas, VA). Cells were cultured in RPMI-1640 supplemented with fetal bovine serum (FBS) 10%, antibiotics (penicillin 100 units/mL; streptomycin 100 *μ*g/mL), and l-glutamine (2 mM) at 37°C in an atmosphere of 5% of CO_2_.

### 2.3. Proliferation Assay

The effect of drug on cell proliferation was determined by using TACS 3-(4, 5-dimethylthiazol-2-yl)-2, 5-diphenyltetrazolium bromide (MTT) cell proliferation assay (Trevigen, Gaithersburg). The cells (2,000 per well) were incubated with or without morphine in triplicate in a 96-well plate and then incubated for 2, 4, and 6 days at 37°C. A MTT solution was added to each well and incubated for 2 h at 37°C. An extraction buffer (20% SDS and 50% dimethylformamide) was added, and the cells were incubated overnight at 37°C. The absorbance of the cell suspension was measured at 570 nm using a microplate reader (DAS Technologies, Chantilly, VA). This experiment was repeated twice, and the statistical analysis was performed to obtain the final values.

### 2.4. Wound-Healing Assay

MDA.MB231 cells were seeded at the density of 40 × 10^3^ cells per well into a 6-multiwell plate and cultured in DMEM medium supplemented with 1% FBS. At the time of confluence, cells were incubated in the absence or presence of morphine (1, 10, and 100 *μ*M) for 48 h after a slit made horizontally with a white tip at the center of each confluent well. Cell invasion on the slit of the confluent well was assessed at 0, 24, 48 hours, in each condition, by light microscopy.

### 2.5. Mice

Six eight-week-old female Foxn1^nu/nu^ mice were purchased by Harlan, San Pietro al Natisone, Italy. Mice were housed five for cage in the standard mice plexiglass cages and maintained on a 12 h light : 12 h dark cycle (lights on at 7.00 a.m.) in a temperature-controlled room (22 ± 2°C) and with food and water ad libitum at all times. All the experiments performed on animal models were in compliance with the guidelines for the Care and Use of Laboratory Animals of the National Cancer Institute “Fondazione G. Pascale,” IRCCS. Moreover, all experiments were performed by also following the European Directive 63/2010/UE and the Italian Law (DL 26/2014, authorized by Minister of Health, Italy). This study was carried out in accordance with the recommendations that cover all scientific procedures involving the use of live animals.

### 2.6. Generation of Heterotopic Mouse Model of Breast Cancer and Experimental Protocol

MDA.MB231 breast cancer cells were harvested from subconfluent cultures after a brief exposure to 0.25% trypsin. Trypsinization was stopped with medium containing 10% FBS. The cells were washed once in serum-free medium and suspended in PBS. Only suspensions consisting of single cells, with >90% viability, were used for the injections. A total of 16 female Foxn1^nu/nu^ mice were used in this experiment and maintained in a barrier facility on HEPA-filtered racks. Animals were individually identified using numbered ear tags. All experiments were conducted in a biological laminar flow hood, and all surgical procedures were conducted with strict adherence to aseptic technique. The mice were anesthetized with Avertin solution injected intraperitoneally according to their weight. A suspension of 2,5 × 10^6^ MBA.MB231 cells in 25 *μ*L of PBS 1X/mouse was injected subcutaneously into the right-side flank area of mice. When tumors reached ~30–60 mm^3^, mice were randomized into the following treatment groups (*n* = 4): (a) normal saline (control) and (b) morphine sulphate at 0.714 mg/kg mouse/day for first 15 days and then 1.43 mg/kg mouse/day (equivalent to 50 mg and 100 mg morphine per day, resp., for a 70 kg human). Tumor volumes were monitored once a week by using a digital caliper. Therapy was continued for 4 weeks and animals were sacrificed 2 weeks later. The tumor size was measured using digital caliper, and the tumor volume was estimated by the following formula: tumor volume (mm^3^) = (*W* × *L*) 2 × 1/2, where *L* is the length and *W* is the width of the tumor. Normally distributed data were represented as mean ± S.E.M. Paired *t*-test one-tailed analysis was used to examine the significance of differences among groups (GraphPad Prism 5.0). A probability value with ^*^ *P* < 0.05 and ^**^ *P* < 0.01 was considered to be statistically significant. Fluorescein isothiocyanate- (FITC-) dextran (100 *μ*L) was injected into the tail vein of mice to visualize microvessels within 150 *μ*m (using single-photon microscopy) or ~600 *μ*m (using multiphoton laser-scanning microscopy [MPLSM]) of a tumor/window interface. Half of the tumor tissue was formalin-fixed and paraffin-embedded for immunohistochemistry and routine H&E staining. The other half was snap-frozen in liquid nitrogen and stored at −80°C.

### 2.7. Preparation of Nuclear Extract from Tumor Samples

Breast tumor tissues (75–100 mg/mouse) from control and experimental mice were prepared according to standard protocols. The supernatant (nuclear extract) was collected and stored at −70°C until use. Protein concentration was determined by the Bradford protein assay with BSA as the standard.

### 2.8. Immunohistochemical Analysis for CD31 in Tumor Tissue

Breast cancer tumor samples from controls and treated mice were embedded in paraffin and fixed with paraformaldehyde. After being washed in PBS, the slides were blocked with protein block solution (DakoCytomation) for 20 min and then incubated overnight with polyclonal anti-goat PECAM-1 (1 : 100). After the incubation, the slides were washed and then incubated with biotinylated link universal antiserum followed by horseradish peroxidase-streptavidin conjugate (LSAB+ kit). The slides were rinsed, and color was developed using 3, 3′-diaminobenzidine hydrochloride as a chromogen. Finally, sections were rinsed in distilled water, counterstained with haematoxylin, and mounted with DPX mounting medium for evaluation. Pictures were captured with a Photometrics CoolSNAP CF colour camera (Nikon, Lewisville, TX) and MetaMorph version 4.6.5 software (Universal Imaging, Downingtown, PA).

### 2.9. Western Blot Analysis

Breast tumor tissues (75–100 mg/mouse) from control and experimental mice were minced and incubated on ice for 1 h in 0.5 mL of ice-cold Lysis Buffer (10 mM Tris, ph8.0, 130 mM Nacl, 1% Triton X-100, 10 mM NaF, 10 mM sodium phosphate, 10 mM sodium pyrophosphate, 2 *μ*g/mL aprotinin, 2 *μ*g/mL leupeptin, and 2 *μ*g/mL pepstatin). The minced tissue was homogenized using a Dounce homogenizer and centrifuged at 16,000 ×g at 4°C for 10 min. Western blotting analysis was performed according to standard protocols. *β*-Actin was used as loading control.

## 3. Results

### 3.1. Morphine Enhances the Proliferation of Triple Negative Breast Cancer Cells, by Performing* In Vitro* Assays on MBA

**Figure 1:**
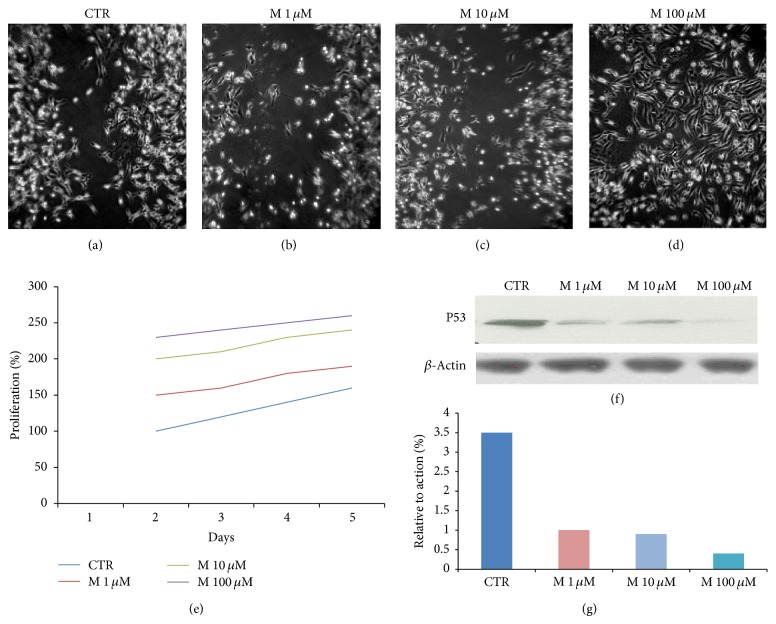
Morphine stimulates proliferation in MDA.MB231 cell lines. MDA.MB231 cells were incubated in medium containing (a) medium control, (b) 1 *μ*m morphine, (c) 10 *μ*m morphine, and (d) 100 *μ*m morphine. Cell migration rates were quantitatively assessed by counting the number of cells in the denuded area at 0, 24, and 48 h after wound induction. At 48 h after wound induction, there were clearly more cells in the denuded area of morphine treated cells than untreated cells. (e) MTT assay results show an enhancement of proliferation in breast cancer cells treated with morphine with respect to control cells. Data are representative of three independent experiments (*P* value < 0.05). (f-g) Western blot showing that morphine reduces the expression of p53 in MDA.MB231 cells treated with morphine (lanes 2, 3, and 4) with respect to controls (lane 1) in dose dependent manner. *β*-Actin was used as loading control.

MB231 breast cancer cells. Wound-healing assay demonstrated that morphine enhances the migration of breast cancer cells at 48 h in dose dependent manner. These results were also confirmed by MTT assay and flow cytometry (Figure 1(e) and data not shown). In order to assess if morphine enhances the apoptosis in breast cancer cells, we performed western blotting analysis of p53 expression on cell lysate extracted from MBA.MB231 cells not treated and treated with morphine. (Figure 1(g)). Taken together, our data showed that morphine inhibits apoptosis and promotes proliferation in dose dependent manner. The same results were also obtained for MCF-7 cells (data not shown).

### 3.2. Morphine Promotes Tumor Growth and Microvessel Formation in Heterotopic Mouse Model of Triple Negative Breast Cancer

**Figure 2:**
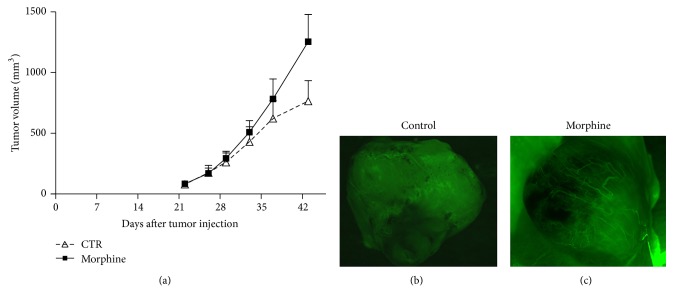
Morphine promotes the tumor growth in TBNC mouse model. (a) Morphine promotes tumor growth in breast tumor xenograft model. Breast tumor growth in mice treated with vehicle (•) and morphine (▪). Tumor volumes increased after 28 days of morphine treatment until 35 days (*P* < 0.05) as compared with control (vehicle-treated). Each point represents the mean of five separate experiments. (b-c) Measurements of fluorescence per second depicting microvessel tumor (FITC-DEXTRANE) using MacroFluo images showed that morphine enhances the angiogenesis in tumor of mice (b) with respect to controls (c).

**Figure 3:**
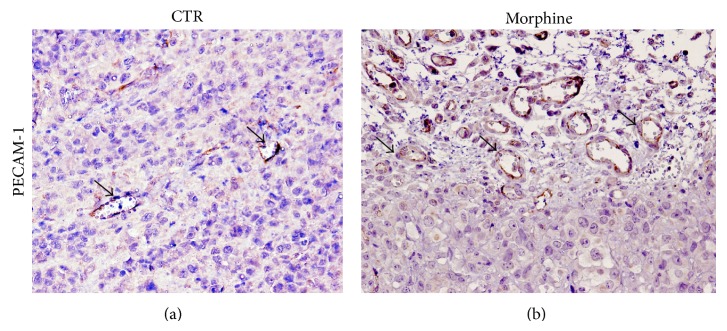
Morphine promotes angiogenesis formation in heterotopic mouse model of breast cancer. Immunohistochemical analysis for PECAM-1showed the enhancement of PECAM-1 expression in morphine treated group (b), compared to controls (a). Arrows indicate positive staining of microvessel staining.

In order to study the role of morphine in the tumor growth* in vivo*, we generated a mouse model of breast cancer by injection of MDA.MB231 cells subcutaneously into the right-side flank area of mice. When tumors reached ~30–60 mm^3^, 2 weeks after cell injection, the mice were randomized into three groups: (a) normal saline (control) and (b) morphine sulphate at 0.714 mg/kg mouse/day for first 15 days and then 1.43 mg/kg mouse/day (equivalent to 50 mg and 100 mg morphine per day, resp., for a 70 kg human). Tumor volumes were monitored once a week by using a digital caliper. Therapy continued for 4 weeks and animals were sacrificed 2 weeks later. We also monitored the body weight of mice twice a week until the end of treatment. No difference was observed between the body weights of two groups of animals, indicating that treatments of mice with drug are not associated with toxicity effects. Mice were sacrificed at the end of treatment. The final tumor volumes on day 35 after the start of treatment showed a significant increase in the morphine group compared with control (Figure 2(a)). Interestingly, administration of morphine enhanced tumor volumes and resulted in rapid growth of tumors with respect to controls. In order to assess if morphine inhibits microvessel formation in breast tumors, fluorescein isothiocyanate- (FITC-) dextran was injected into the tail vein of mice. Our data demonstrate that morphine enhanced microvessel formation in mice tumors with respect to controls (Figures 2(b)-2(c)). In order to confirm these data, we performed an immunohistochemical staining with CD31 on tumor tissues from control and treated mice. Our data demonstrate that morphine promotes microvessel formation in breast tumors of mice treated with respect to controls (Figures 3(a)-3(b)).

## 4. Discussion 

Several experimental studies performed in* in vitro* and* in vivo* cancer cell lines and mouse models showed that morphine plays a role in regulation of cancer cell growth and metastasis. The results obtained by these studies are still controversial since many reports showed that morphine was able to inhibit the growth of various human cancer cell lines [6–12] or animal models [13–16]. On the contrary, other studies proved that morphine increased tumor cell growth in* in vivo* [17, 18] or* in vitro* [19] models. To study cancer cell growth promoting or inhibiting effects of morphine, several xenograft mouse models were generated. Tegeder et al. [13] generated a mouse model of breast cancer by subcutaneous injection of MCF-7 and MDA-MB231 cells in NMRI-nu/nu mice. In this paper, it has been demonstrated that morphine significantly reduced tumor growth through a p53-dependent mechanism. Additionally, in these mice, naloxone increased the growth-inhibitory effects of morphine. Similar results were obtained in rat model of colon cancer in which subcutaneous administration of morphine leads to significant decrease in the hepatic tumor burden. On the contrary, several experimental studies demonstrated that morphine increased tumor growth. Gupta et al. in orthotropic mouse model of breast cancer obtained by injection of MCF-7 cells into the mammary fat pad of nude mice demonstrated that morphine, in clinically relevant doses, increased tumor growth. This was associated with increased angiogenesis and inhibition of apoptosis and promotion of cell cycle progression [20]. In this study, it was also reported that naloxone itself had no significant effect on angiogenesis. According to these results, in another study, it was demonstrated that morphine, subcutaneously administrated in mice, increased the tumor growth in mouse model of leukaemia and sarcoma. In these mice, morphine had also a general immunosuppressive effect [25].

These contrasting results are probably associated with different concentration and/or time of administration of morphine. In fact,* in vitro* and* in vivo* studies demonstrated that tumor-enhancing effects with morphine occur after administration of low daily doses or single dose of morphine [22], while tumor suppression occurs after chronic high doses of morphine [11, 15, 16].

Thus, there is a dilemma about the effects of morphine on cancer cell growth and angiogenesis in various types of cancer [23]. The role of morphine in the regulation of tumor cell growth is not yet correctly established.

It has also been demonstrated that the *μ*-opioid receptor, by which morphine exerts its action, directly regulates tumor growth and metastasis. On the basis of these results, different mechanisms of opioid receptor-mediated influence of morphine on tumor growth have been proposed. Morphine, as mentioned above, after binding to the *μ*-opioid receptor, regulates cell cycle progression by stimulating mitogen-activated protein kinase (MAPK)/extracellular growth factor (Erk) pathways [20]. Alternatively, morphine can mediate apoptosis by activating phosphatidylinositol 3-kinase (PI3K)/protein kinase B (Akt) pathway [26]. Additionally, morphine by upregulation of urokinase plasminogen activator (uPA) expression induces metastasis formation [27], while by transactivation of VEGF receptor, it induces angiogenesis [28]. Finally, morphine affects also the function of T lymphocytes, leading to immunosuppression [29].

It has been proposed that morphine plays also a role in tumor apoptosis. Apoptosis is a form of cell death in which a programmed sequence of events leads to the elimination of cells without releasing harmful substances into the surrounding area. It is noted that apoptosis is regulated by two pathways: the mitochondrial-mediated pathway (intrinsic) [30] and death receptor-mediated pathway (extrinsic) [31]. It is noted that, in cancer cells, apoptosis is deregulated, and this leads to quick proliferation and tumor growth [32, 33]. Morphine was shown to induce apoptosis of macrophages, T lymphocytes, and human endothelial cells [34, 35]. Experiments performed on human tumor cell lines demonstrated that morphine in high concentration induces apoptosis and inhibits cancer cell growth by activation of different signal pathways involving caspase 3/9 and cytochrome c, sigma-2 receptor. Additionally in SH-SY5Y cells, morphine has antiapoptotic effect by antagonizing doxorubicin [36]. These discrepancies, also in these cases, are associated with different cell line tumor type used and/or* in vivo* dose/time of morphine administrated.

Recent data demonstrated a role of morphine in angiogenesis. Angiogenesis is required for invasive tumor growth and metastasis and represents an important point in the control of cancer progression. Proangiogenic activity of morphine was demonstrated in the MCF-7 breast cancer model. In these mice, morphine at clinically relevant concentrations enhanced tumor neovascularization [20]. In an animal model of hormone-dependent breast cancer, it has also been demonstrated that morphine promoted activation of vascular endothelial growth factor (VEGF) receptor and increased metastasis [21, 29]. It has been proposed that morphine explains its proangiogenic activity by stimulation of mitogen-activated protein kinase (MAPK) signalling pathway via G protein-coupled receptors and nitric oxide (NO). Alternatively, several* in vivo* studies provided evidence that morphine can induce tumor growth by upregulation of cyclooxygenase-2 (COX-2) [37–40] and or prostaglandin E2-mediated stimulation of angiogenesis [41–44]. On the contrary, several* in vivo *and* in vitro* studies demonstrated that morphine can inhibit angiogenesis by regulation of different pathways [8, 34, 45, 45–52]. These different results can be due to different experimental conditions (cell line tumor type used and/or dose/time of morphine). Morphine plays a role not only in tumor cell growth but also in metastasis formation, which is the main process related to most cancer deaths and failure in cancer treatment [53, 54].

For these reasons, in order to elucidate the role of morphine in regulation of tumor growth and angiogenesis in breast cancer we performed* in vitro* and* in vivo *studies on the MDA.MB231 breast cancer cells. These cells are triple negative (basal-like) breast cancer (TNBC) cells. It is noted that TNBC does not express the estrogen receptor (ER), progesterone receptor (PR), or human epidermal growth factor receptor type 2 (HER2). Due to lack of both hormone receptors and HER2 expression, patients with this type of breast cancer have no chance to benefit from the endocrine therapy and HER2 targeted therapy. In addition, we used immunodeficient mice that are able to mimic the compromised immune system of a patient with breast cancer. So in these experimental conditions, it has become interesting for us to study the role of morphine in the regulation of cancer cell growth and angiogenesis. Our data showed that morphine in TNBC at clinical relevant doses promotes tumor angiogenesis and increases breast cancer progression. For these reasons, it is very important for the management of severe pain associated with cancer to consider accurately the dose and route of administration of morphine in order to avoid severe effect of cancer progression. Further studies are ongoing in our laboratory in order to dissect the molecular mechanisms underlying the role of morphine in cancer development and metastasis formation in breast cancer. Specifically, we will generate orthotropic mouse models of breast cancer by using not only MDA.MB231 cells but also MCF-7 (human breast adenocarcinoma cell line) cells which represent estrogen receptor (ER) positive control cell lines. The results obtained from these data will shed light on the role of morphine in regulation of breast cancer progression.
